# Determinants of recent HIV testing among male sex workers and other men who have sex with men in Shenzhen, China: a cross-sectional study

**DOI:** 10.1186/1471-2334-14-S2-P19

**Published:** 2014-05-23

**Authors:** Rui Cai, Jin Zhao, Wende Cai, Lin Chen, Jan Hendrik Richardus, Sake J de Vlas

**Affiliations:** 1 Erasmus MC, University Medical Center Rotterdam, Rotterdam, the Netherlands

## Background

Men who have sex with men (MSM), including male sex workers (also known as money boys, MBs), are highly vulnerable to HIV, but HIV testing rates remain unacceptably low in China. We compared and separately evaluated determinants of recent HIV testing among MBs and other MSM.

## Methods

We recruited 510 MBs and 533 other MSM in Shenzhen by time-location sampling. We compared their HIV testing history in the last year, socio-demographics and behavioral information, and we performed logistic regression to identify determinants of having been tested in the last year among MBs and other MSM.

## Results

Compared to other MSM, MBs had a slightly lower rate of HIV testing (43% versus 48%, p = 0.10). MBs having multiple anal sex partners (adjusted odds ratio, AOR = 2.03, p=0.006) and having more commercial male partners (> 4 versus ≤4 commercial male partners, AOR = 1.49, p = 0.053) reported a higher rate of HIV testing. Condom use was not associated with HIV testing among MBs. Among other MSM, HIV testing was reported more often for those with homosexual orientation than for those with bisexual orientation (58% versus 37%, p = 0.003) and for those with heterosexual orientation (58% versus 28%, p = 0.018). Other MSM who had only male sex partners (AOR = 1.82, p = 0.026), and those having a STI history (AOR = 2.53, p = 0.004) reported more often HIV testing. Living in Shenzhen longer was positively correlated with HIV testing for both MBs (p = 0.078) and other MSM (p < 0.001).

## Conclusion

Compared to Western countries, the HIV testing rates among MBs and other MSM are still low in Shenzhen, China. For MBs, testing should be advocated through education programs to provide better HIV and condom use knowledge. For other MSM, de-stigmatizing programs are urgently needed to encourage those with female sex partners to go for testing services. For both MSM groups, more efforts are needed to target the new comers.

